# Optimization of a Sonotrode Extraction Method and New Insight of Phenolic Composition of *Fucus vesiculosus*

**DOI:** 10.3390/md23010040

**Published:** 2025-01-14

**Authors:** Lidia Gil-Martínez, Alejandro Santos-Mejías, José Manuel De la Torre-Ramírez, Alberto Baños, Vito Verardo, Ana M. Gómez-Caravaca

**Affiliations:** 1 Department of Chemistry and Natural Products, DMC Research Center, Camino de Jayena s/n, 18620 Alhendín, Spain; lidiagm@dmcrc.com (L.G.-M.); josemanuel.delatorre@dmcrc.com (J.M.D.l.T.-R.); 2 EpiChron Research Group, Aragon Health Sciences Institute (IACS), IIS Aragón, Miguel Servet University Hospital, 50009 Zaragoza, Spain; asantosm@ext.aragon.es; 3 Department of Microbiology, DMC Research Center, Camino de Jayena s/n, 18620 Alhendín, Spain; abarjona@dmcrc.com; 4 Department of Nutrition and Food Science, University of Granada, Campus of Cartuja s/n, 18071 Granada, Spain; 5 Institute of Nutrition and Food Technology ‘José Mataix’, Biomedical Research Center, University of Granada, 18071 Granada, Spain; anagomez@ugr.es; 6 Department of Analytical Chemistry, University of Granada, Campus of Fuentenueva s/n, 18071 Granada, Spain

**Keywords:** brown seaweeds, Box–Behnken design, chlorophenols, bromophenols, antioxidant capacity, sulfated phenols

## Abstract

The optimization of bioactive compound extraction from *Fucus vesiculosus* using ultrasound-assisted extraction (UAE) via sonotrode was investigated to maximize phenolic recovery and antioxidant activity while promoting a sustainable process. Optimal conditions (40% *v*/*v* ethanol in water, 38 min, 36% amplitude) were selected to maximize phenolic recovery while considering environmental and energy sustainability by optimizing extraction efficiency and minimizing solvent and energy usage. HPLC-ESI-QTOF-MS analysis tentatively identified 25 phenolic compounds, including sulfated phenolic acids, phlorotannins, flavonoids, and halophenols, with some reported for the first time in *F. vesiculosus*, underscoring the complexity of this alga’s metabolome. The antioxidant activity of the optimized extract was evaluated through FRAP (143.7 µmol TE/g), DPPH (EC_50_ 105.6 µg/mL), and TEAC (189.1 µmol Trolox/g) assays. The optimized process highlights *F. vesiculosus* as a valuable source of natural antioxidants, with potential applications in biotechnology, cosmetics, and food industries.

## 1. Introduction

Seaweeds are a diverse group of macroscopic, multicellular, photosynthetic aquatic organisms comprising thousands of species. They are categorized into three taxonomic groups based on the pigmentation of their tallus: Phaeophyta (brown algae), Chlorophyta (green algae), and Rhodophyta (red algae) [1]. *Fucus vesiculosus*, a brown alga, belongs to the family Fucaceae, which encompasses five genera: *Ascophyllum*, *Fucus*, *Pelvetia*, *Pelvetiopsis*, and *Silvetia* [2]. Among these, the genus Fucus has the widest global distribution, predominantly found in high-salinity waters at depths of 0.5–4 m in the Northern Hemisphere, including the Baltic Sea, North Sea, Pacific and Atlantic Oceans, and the Canary Islands [2,3]. *F. vesiculosus* is characterized by a root-like holdfast, a slender stipe, and flat forked blades. Its blades have multiple pairs of air bladders (vesicles) that enable the plant to float vertically underwater [4].

Brown macroalgae, including *F. vesiculosus*, are notable for their high content of unique phenolic compounds called phlorotannins (PTs). Additionally, they can be a source of phenolic acids, flavonoids, phenolic terpenoids and mycosporine-like amino acids [5,6]. These algae are also sources of pigments (e.g., fucoxanthin) and bioactive polysaccharides, including fucoidans, laminarans, and alginates [7]. Phlorotannins, specifically, are oligomers of phloroglucinol (1,3,5-trihydroxybenzene) linked in various ways, with molecular sizes ranging from 126 Da to 650 kDa [8]. Depending on the linkages, phlorotannins are classified into several types: fuhalols, phlorethols, fucols, fucophlorethols, eckols, and carmalols [1]. These compounds contribute to cell wall biosynthesis and serve as defense mechanisms, acting as herbivore deterrents, digestive inhibitors, and antibacterial and antifouling agents [2,9].

Sulfated metabolites, particularly sulfated phenolic derivatives, are frequently found in seaweeds and marine organisms. These compounds often function as precursors to bioactive substances, with diverse ecological roles [10,11]. Sulfation enhances the hydrophilic nature and aqueous solubility of molecules, thereby increasing their bioavailability. It may also influence the pigmentation of seaweed biomass by stabilizing complexes with other chromophores. Furthermore, it is hypothesized to play a role in the neutralizing of toxic compounds and in the modulation of plant growth processes [12,13]. Interestingly, it can also be found in terrestrial plants [14].

In contrast, bromophenols are uniquely marine, as bromination occurs predominantly in organisms with access to bromides in seawater. This process is facilitated by the enzyme vanadium bromoperoxidase, which catalyzes the bromination of organic substrates in the presence of bromide ions and hydrogen peroxide [15]. These brominated molecules are thought to be responsible for the typical sea-like taste and flavor of seafood [16]. Structurally, bromophenols are categorized into simple bromophenols, bromophenol derivatives, and highly brominated mono- and bis-phenols [17] that contain one or more phenolic rings, bromine, and different substituent radicals [1]. While primarily associated with red seaweeds [18], bromophenols have also been detected in brown algae such as *Padina arborescens*, *Sargassum siliquastrum*, and *Lobophora Variegata* [15].

These phenolic compounds demonstrate a wide range of health benefits, including antioxidant, antimicrobial, anti-inflammatory, and anticancer properties. They also exhibit anti-diabetic, antihypertensive, antihyperlipidemic, and anti-obesity effects [19,20,21].

The abundance of phenolic compounds in *F. vesiculosus* extracts is influenced by environmental factors such as temperature, season, pollutants, and sunlight, impacting the richness of the extract [22]. Extraction conditions, such as drying temperature, solvent choice, and extraction method, also play a significant role. Low drying temperatures (35–50 °C) help preserve phenolic compounds, compared to higher temperatures (>65 °C), which can cause degradation. Solvent polarity significantly impacts phenolic extraction yield, with ethanol being preferred for its low toxicity [23]. Pre-treatment with low-polarity solvents like hexane, acetone, or dichloromethane reduces interference from pigments and fatty acids. While traditional extraction methods (e.g., soxhlet, maceration, percolation) have limitations, non-traditional methods such as ultrasound, microwave, and supercritical fluid extraction offer improved efficiency and environmental benefits [4,22,24]. Ultrasound-assisted extraction, in particular, disrupts cell walls using mechanical waves, enhancing compounds release [25]. This technique leverages acoustic cavitation—the creation, expansion, and collapse of microbubbles caused by ultrasonic waves—to drive extraction through phenomena like compression, rarefaction, pressure, agitation, and radical formation [26]. In addition, ethanol–water mixtures are less toxic and more sustainable compared to pure organic solvents.

This study aims to optimize the recovery yield of phenolic compounds while minimizing energy consumption, extraction time, and the use of organic solvents, thereby improving the sustainability of the procedure. To achieve this, a solid–liquid extraction model assisted by low-amplitude ultrasound (20–40%) was developed and optimized using Box–Behnken statistical design. Furthermore, chemical profiling via HPLC-ESI-QTOF-MS of the extract confirmed the presence of 25 phenolic compounds, including well-known phenolic compounds such as phlorotannins, as well as 11 compounds reported for the first time in *F. vesiculosus*, such as sulfur-containing phenols and halophenols (bromo- and chlorophenols). Additionally, the antioxidant activity of the extract was evaluated, underscoring its potential applications in the food industry.

## 2. Results and Discussion

### 2.1. Fitting the Model

The sonotrode UAE parameters were optimized using a Box–Behnken experimental design, detailed in Table 1.

Experimental TPC values ranged from 0.71 ± 0.06 to 2.93 ± 0.09 mg GAE/g dry seaweed. The highest TPC recovery was achieved using 50% ethanol as the solvent with an extraction time of 40 min at 40% amplitude. In contrast, the lowest recovery was observed when 90% ethanol was used as the solvent, with a time of extraction of 2 min and 30% amplitude.

The data presented in Table 1 were used to analyze the combined effects of ethanol/water ratio, extraction time, and amplitude on TPC recovery during UAE.

Table 2 details the model’s regression coefficients and the analysis of variance (ANOVA) results. The model was evaluated according to the significance of the regression coefficients, quadratic correlation coefficients (R^2^), and lack of fit. Following the ANOVA test, the model was recalculated, excluding the non-significant terms with a significance level of *p* > 0.05.

Significant interactions affecting the TPC response variable included the linear effects of ethanol/water ratio (β_1_), time (β_2_), and amplitude (β_3_), as well as the crossed interaction between ethanol/water ratio and time (β_12_), the quadratic effect of ethanol/water ratio (β_11_), and the quadratic effect of amplitude (β_33_).

The ANOVA test confirmed the validity of the model, indicating a significant regression model (*p* < 0.05) and a non-significant lack of fit for the response variable (*p* > 0.05).

### 2.2. Analysis of Response Surfaces

**Figure 1:**
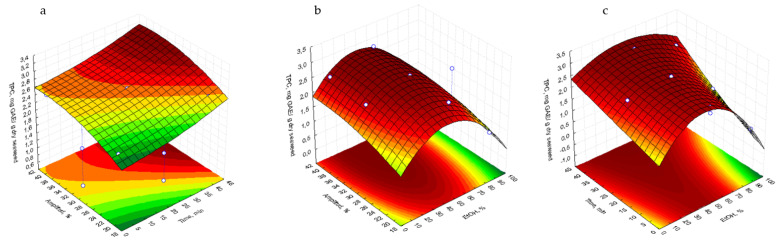
Response surface plots showing combined effects of process variables for TPC (mg GAE/g dry seaweed.): amplitude (%)–time (min) (**a**), amplitude (%)–% EtOH (**b**), and % EtOH–time (min) (**c**); GAE: Gallic acid equivalents.

The optimal extraction conditions were identified by analyzing the response surface plots that illustrate the interactions between % EtOH (X1) and time of extraction (X2) as well as % amplitude (X3) (Figure 1). The 3D surface plots were used to represent each pair of variables, with the remaining variable held constant at its central level.

The surface response plots for TPC indicated that the highest responses were achieved with 36–40% amplitude and 35–40 min of extraction (Figure 1a), 30–40% amplitude and 20–60% ethanol (Figure 1b), and 30–60% ethanol with sonication for 35–40 min (Figure 1c).

### 2.3. Optimization of Sonotrode Parameters

Determining the optimal conditions is the final step after analyzing the 3D plots of the RSM. The optimal conditions to maximize the extraction of phenolic compounds from *F. vesiculosus* are summarized in Table 3. The model’s accuracy was validated by comparing the predicted values with the experimental results.

The optimal extraction conditions were 40% ethanol/water (*v*/*v*), 38 min of extraction, and 36% amplitude. Ethanol was demonstrated to be inefficient when used pure or in high concentration, probably due to the solvation provided by the water on the mixture and phlorotannins’ polarity, as they are generally more soluble in water [27,28]. The optimal extraction time chosen was the shortest duration that provided maximum efficiency in the extraction of the antioxidant compounds, aiming to achieve an environmentally friendly process that is both fast and energy-efficient in comparison with traditional methodologies. The accuracy of the mathematical model was verified through the extraction of bioactive compounds from *F. vesiculosus* dry material, using the optimal conditions. The experimental values obtained were not significantly different from the predicted values, showing coefficients of variation lower than 10 for TPC assay (briefly, any significant difference *p* < 0.05 between predicted and experimental data was noticed).

Regarding the optimization of the extraction of bioactive compounds from *F. vesiculosus* using UAE, Golshany et al. [28] focused on the extraction of phlorotannins, reporting a yield of 9.54 mg of phloroglucinol equivalents (PGE)/g d.w. using water as the solvent and a 10 min treatment at a sonication power of 33%. Their study compared the effectiveness of low sonication power (33% the lowest tested value) against higher intensity levels of 66.5% or 100%, finding no significant differences in the phlorotannin recovery yields. In this case, sonicaction power was expressed as a percentage of the 1200 W available as the total ultrasonic power. Similarly, Ummat et al. [26] found that although the application of ultrasound significantly improved the extraction yield of phenolic compounds (35 kHz and 130 kHz), higher US frequencies did not significantly improved the extraction yields. They reported optimum yields of 571.1 mg GAE/g of dry *F. vesiculosus* extract using 35 kHz US frequency in 50% ethanol for 30 min. Consistent with these findings, and aiming to innovate within the field, we investigated the effect of low amplitude sonication (ranging from 20 to 40%) in the extraction of phenolic compounds from *F. vesiculosus*. However, our statistical analysis revealed significant differences in the phenolic recovery yield at these low amplitudes, with the optimal range identified between 36 and 40% amplitude. In contrast, Garcia-Vaquero et al. [24] obtained yields of 316.33 mg dry *F. vesiculosus* extract/g d.w. using UAE at 100% amplitude for a duration of 10 min. with 50% ethanol as the solvent. And Obluchinskaya et al. [29] optimized phlorotannin extraction from *F. vesiculosus* using NADES as the solvent, achieving an optimal yield of extraction of 137.3 mg PGE/g d.w. with 30% water as the solvent, 22.8 min of extraction, and sonication in a bath at 42 kHz.

Considering these results, the variability in the recovery rates of dry material, TPC, and other significant phytochemicals across different studies is remarkable. This variation may be attributed not only to the impact of technological treatments but also to the initial concentration of these bioactive compounds in the biomass, as it is well established that the levels of bioactive compounds in macroalgae can be significantly affected by factors such as geographical location and the season in which they are harvested [30].

### 2.4. HPLC-ESI-TOF-MS Tentative Profiling of Phenolic Compounds in the Optimized F. vesiculosus Extract

Phenolic compounds were characterized using a non-targeted mass spectrometry approach through LC-ESI-QTOF-MS analysis in negative ionization mode. They were tentatively identified based on their experimental and calculated *m*/*z* values, retention times, mass error (ppm), scores (%), molecular formulas, predominant in-source fragment ions, and isotopic distribution.

In total, 25 phenolic compounds were tentatively identified, including 2 phenolic acids, 15 phlorotannins, 1 flavonoid, and 7 halophenols (Table 4). To the best of our knowledge, this is the first research article describing halophenols and sulfated phenolic compounds in the brown seaweed *F. vesiculosus*.

#### 2.4.1. Phenolic Acids

**Figure 2:**
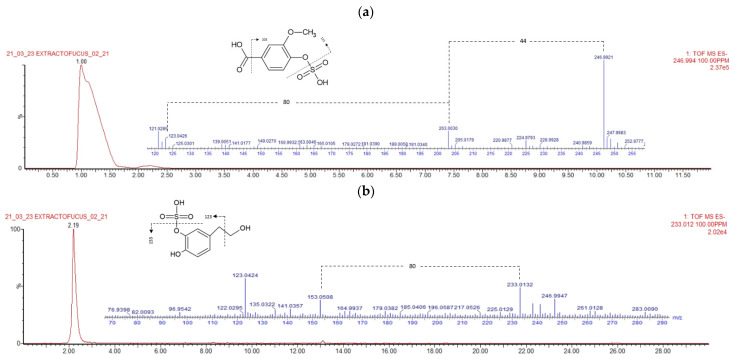
(**a**) Peak MS/MS spectra of tentatively identified vanillic acid sulfate, showing fragment ions *m*/*z* 247, *m*/*z* 203, and *m*/*z* 123 and presumed fragmentation pattern of the molecule. (**b**) Peak MS/MS spectra of tentatively identified hydroxytyrosol sulfate, showing fragment ions *m*/*z* 233, *m*/*z* 153, and *m*/*z* 123 and presumed fragmentation pattern of the molecule.

Two phenolic acids were identified in this study, both of which were sulfated derivatives. Compound 1 (rt = 1.101), with [M-H]^−^ *m*/*z* 247, was tentatively characterized as Vanillic acid 4-sulfate (C_8_H_8_O_7_S) based on the formula score and the mass fragmentation pattern (Figure 2a). As we can observe, the MS/MS spectra of the peak reports one fragment with *m*/*z* 203, which may correspond to the loss of the carboxylic acid group (−44 uma), and a fragment 123, corresponding with the leakage of the sulfated residue from the molecule (−80 uma), which is consistent with the previous literature [31]. Vanillic acid 4-sulfate has previously been identified in other seaweeds such as *Ulva* sp. [31,32], *Sargassum* sp., and *Centroceras* sp. [32], but not in *F. vesiculosus*. Compound 3 (rt = 2.206), with *m*/*z* 233, was tentatively identified as hydroxy tyrosol sulfate (C_8_H_10_O_6_S). As we can see in Figure 2b, the MS/MS spectra of the molecule reveal a fragment with *m*/*z* 153, corresponding to that of the hydroxytyrosol molecule after the release of the sulphated residue (−80 uma). Furthermore, ion *m*/*z* 123 is characteristic of the fragmentation of hydroxytyrosol by MS spectrometry, and the mass spectra correlates with the characterization of the molecule in other matrixes [33]. This is the first time that hydroxy tyrosol sulfate has been reported in seaweeds. However, Norskov et al. identified tyrosol 4-sulfate in some of the red and brown seaweeds they analyzed in their research [31], and Zhong et al. identified hydroxytyrosol 4-O-glucoside in *Centroceras* sp., *Dasya* sp., *Grateloupia* sp., and *Sargassum* sp. algae [32].

#### 2.4.2. Phlorotannins

Fourteen phlorotannins with a number of phloroglucinol monomeric units (PGUs) ranging from 4 to 10 have been detected in this study. All of them had previously been identified in *F. vesiculosus*. Peak 2, with an *m*/*z* value of 497 and a molecular formula of C_24_H_18_O_12_, was identified as fucodiphlorethol, a tetramer. The mass spectrum showed fragmentation ions at *m*/*z* 353, corresponding to a loss of 1PGU and water, and *m*/*z* 230, corresponding to a loss of 2 PGU and water. Additionally, a fragment at *m*/*z* 124 corresponding to phloroglucinol was observed, according to the previous literature [29,34,35]. Peaks 4 (rt = 3.212), 5 (rt = 3.402), 8 (rt = 5.401), and 9 (rt = 5.645) were identified as phloroglucinol hexamers with an *m*/*z* of 745 and a molecular formula of C_36_H_26_O_18_. Based on bibliographic data and fragmentation patterns, these compounds likely correspond to different fucophlorethol isomers. These phlorotannins showed fragment losses of one or more PGU through the leak of ether bonds, with losses of 124 and 166 Da observed, consistent with the data and results of previous studies [29,34]. Due to the structural complexity and diversity of phlorotannins, further analytical techniques are required to confirm these identifications. Peaks 10 and 11 (*m*/*z* 869) were tentatively identified as fucophlorethol heptamers (C_42_H_30_O_21_). Their fragmentation patterns were similar, displaying fragment ions at *m*/*z* 725, corresponding to the loss of phloroglucinol and water (166 Da) via ether bond cleavage. The same fragmentation pattern was observed for compounds 12, 13, and 14, with an *m*/*z* of 933 and a molecular formula of C_48_H_34_O_24_ tentatively identified as fucophlorethol octamers. Peaks 16 and 17, with *m*/*z* 1241 and 1117, respectively, were characterized as fucophlorethol decamer (C_60_H_42_O_30_) and fucophlorethol nonamer (C_54_H_38_O_27_), respectively [28,29,34,35,36].

Peak 15 (rt = 9.385 min) gave *m*/*z* = 591 and a molecular formula of C_24_H_16_O_16_S with a score of 100%. This formula was tentatively identified as Diphlorethohydroxycarmalol sulphate, with fragmentation yielding fragments of 511 (related to the leakage of SO_3_) and 385 (additional loss of PGU, +1), according to the work developed by Allwood et al. (2020), in which they could identify this molecule in *Ascophyllum nodosum* [36]. To our knowledge, this molecule has not been described before in *F. vesiculosus*.

#### 2.4.3. Flavonoids

One flavonoid has been identified in the extract. Peak 19 (rt= 10.883) with *m*/*z*= 317 and a molecular formula of C_15_H_10_O_8_ was tentatively characterized as Myricetin, which was previously identified in other brown seaweeds such as *Turbinaria ornate* but not in *F. vesiculosus* [37].

#### 2.4.4. Halophenols

Seven halophenols were detected in the *F. vesiculosus* extract: three brominated phenols and four chlorinated phenols. Halogenated compounds are relatively straightforward to identify by mass spectrometry due to the distinctive isotopic patterns of bromine and chlorine. Chlorine isotopes (masses 35 and 37) display a characteristic natural abundance ratio of 3:1, while bromine isotopes (masses 79 and 81) show a 1:1 ratio. Molecules containing two bromine atoms can be identified by their characteristic molecular ion abundance pattern, which appears in a 1:2:1 ratio at two-mass-unit intervals [38,39,40]. Molecules with two or more chlorine atoms can be easily identified as well, thanks to their isotope patterns. Molecules with two chlorine atoms present an isotope pattern 9:6:1, molecules with three chlorine atoms have one of 27:27:9:1, and with four chlorine atoms this is 54:81:18:3:1 [40].

**Figure 3:**
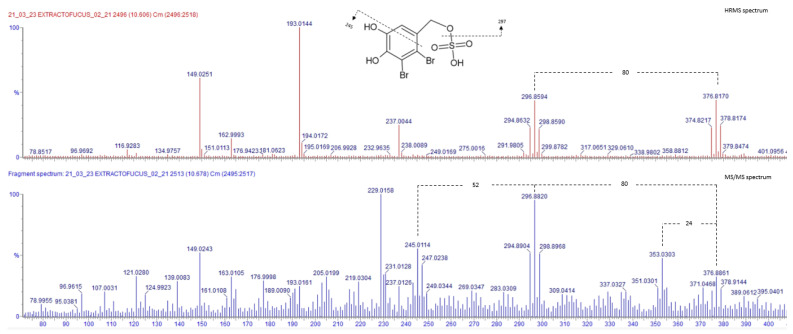
HRMS spectra of Peak 18, with the proposed molecule of lanosol sulfate indicating the *m*/*z* of the fragments (in red), and MS/MS spectrum (in blue).

As shown in Figure 3 in red, the HRMS of Peak 18 reveals two ion fragments containing two bromine atoms each: one ion with *m*/*z* 375, 377, and 379, and a fragment with *m*/*z* 295, 297, and 299. The neutral loss of 80 uma between these fragments suggests the detachment of a sulfate group (SO_3_), because the number of bromine atoms in the molecule remains unchanged. This specific loss of SO_3_ is typical of compounds where sulfate is attached to carbon via oxygen (-C-O-SO_3_H) [14].

In the MS/MS spectrum of the Peak 377 (Figure 3 in blue), no additional brominated fragments were observed to support further structural elucidation. A fragment with *m*/*z* 243, 245, and 247 might indicate the loss of SO_3_ and C_3_H_3_O. Furthermore, the increase in intensity of an ion 96.9615 may correspond to SO_4_H, indicating the presence of sulfated molecules in the spectrum. No information about the molecular ion 377 was found in the literature. But given that ions at *m*/*z* 377 and 297 may differ by a sulfate group, the tentative molecular formula for the 297 fragment was proposed as C_7_H_6_O_3_Br_2_, based on HRMS data (observed *m*/*z* 294.8890, calculated for C_7_H_6_O_3_^79^Br_2_: 294. 8605). This molecular formula corresponds to the bromophenol lanosol (2,3-dibromo-4,5-dihydroxybenzyl alcohol), a compound that is well documented and has previously been identified in red seaweeds such as *Odonthalia corymbifera* [41], *Osmundaria colensoi* [42], and *Vertebrata lanosa* (formerly *Polysiphonia lanosa*) [43,44]. Therefore, the compound eluting at a retention time of 18.618 min. is tentatively assigned the molecular formula C_7_H_6_O_6_SBr_2_, corresponding to lanosol sulfate (a bromophenol not previously reported in seaweeds to our knowledge, although similar molecules such as lanosol-4,7-disulfate have been identified in *V. lanosa* [44]). It should be noted that the unambiguous characterization of the molecule requires additional analytical techniques, such as NMR spectroscopy.

**Figure 4:**
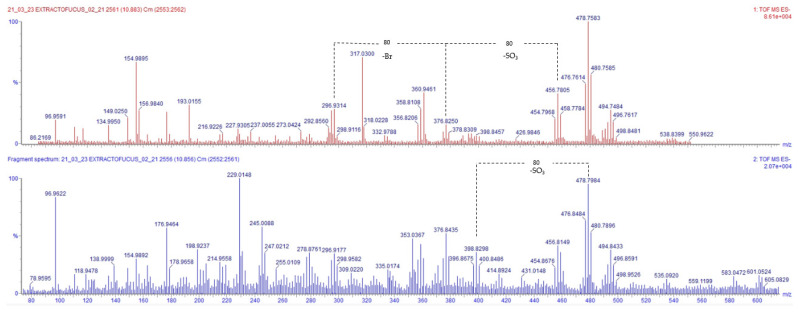
HRMS and MS/MS spectra at retention time of 10.883, indicating the proposed fragments for Peak 20 (in red) and the tentative fragments for Peak 21 (in blue).

Peaks 20 and 21 with *m*/*z* 477, 479, and 481 and 455, 457, and 459, respectively, coeluted at a retention time of 10.883 min., and may correspond to sulfated bromophenols. As can be observed in Figure 4, HRMS and the MS/MS spectra of the 479 ion show losses of −80 uma without altering the isotopic pattern of the fragments, which may correspond to the loss of SO3. Furthermore, a fragment that may correspond with the loss of a bromine atom from the structure of the molecule with *m*/*z* 376, showing an ion with *m*/*z* 295 and 297 with a fragmentation pattern 1:1, can be observed. Due to the complexity of the spectra, it is difficult to differentiate among the fragments that may correspond to the fragmentation of a single molecule from the fragments corresponding to different molecules. Furthermore, there are very few research articles describing the identification of halophenols through mass spectrometry. This makes it very difficult to be accurate in the tentative identification of these kinds of molecules.

**Figure 5:**
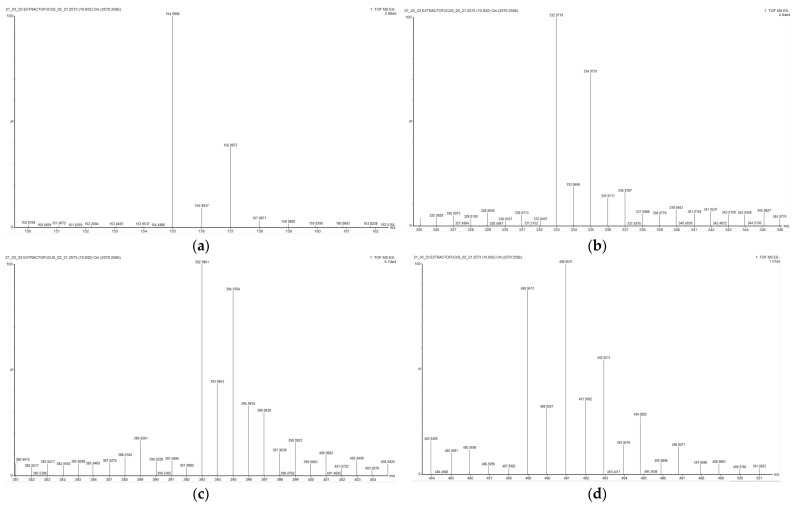
HRMS of compound 23, tentatively identified as chlorobenzoic acid (**a**), compound 24, dichlorophenol (**b**), compound 25, trichlorophenol (**c**), and compound 26, tetrachlorophenol (**d**).

Additionally, at a retention time of 10.932 (peak 22), an ion with *m*/*z* = 154.9906 and 156.9874 (isotopic pattern 3:1) that may correspond to chlorobenzoic acid can be observed. In addition, at a retention time of 10.957, various ion fragments corresponding to chlorophenols with different numbers of chlorine atoms in their molecules appear on the mass spectrum: one dichlorophenol (Peak 23), one trichlorophenol (Peak 24), and one tetrachlorophenol (Peak 25), with their characteristic isotopic patterns (Figure 5) [40].

### 2.5. Antioxidant Activity of F. vesiculosus Extract

The antioxidant activity of the extract was evaluated using the FRAP, DPPH, and TEAC assays. Results are presented in Table 5.

These findings are comparable to results reported in previous studies, although certain variations are observed. For example, Duan et al. obtained *F. vesiculosus* extracts using ultrasound-assisted extraction and various solvents including water, methanol, ethanol, acetone, and ethyl acetate. The best results were obtained using methanol as a solvent, with FRAP and TEAC values of 101.24 mmol TE/ g d.w. and 217.6 mmol TE/ g d.w., respectively. Ethanolic and aqueous extract yielded comparable outcomes, with FRAP values of 108.2 and 111.7 mmol TE/ g d.w. and TEAC values of 177.8 and 171.9 mmol TE/g, respectively [45]. Coelho et al. [19] obtained an aqueous *F. vesiculosus* extract through solid liquid extraction aided by heating up to 121 °C. This extract, used at a concentration of 250 mg/mL, achieved a 20.8% inhibition of oxidation. This inhibition is notably lower than the antioxidant activity suggested by our result, further highlighting the efficiency of our extraction method using ultrasounds and a mixture of ethanol–water as the solvent.

On the other hand, Agregán et al. reported an aqueous *F. vesiculosus* extract obtained with ultrasounds with an ABTS value of 1046.79 mmol TE/g, an EC_50_ of 4.19 mg extract/mL, and FRAP value of 51.66 mmol TE/g extract [46]. While their ABTS results significantly surpassed the TEAC value observed in our study, and the EC_50_ was also better, the FRAP value reported by Agregán et al. was considerably lower, possibly reflecting differences in compound solubility and extraction efficiency. Meanwhile, Getachew et al. prepared an *F. vesiculosus* extract using accelerated solvent extraction (ASE), and the optimal extracts presented EC_50_ values of 140 mg/mL for their *F. vesiculosus* extracts [4]. Finally, Sumampow et al. prepared ethanolic extracts through the pressurized liquid extraction (PLE) of *F. vesiculosus* with an EC_50_ of 92.6 mg/mL [47].

The differences in the antioxidant activity of the extracts underscore the variability of results stemming from differences in extraction methods, solvents, assay conditions, and possible synergistic effects among the compounds present in the extracts [19]. Additionally, it is important to highlight the variability in the concentrations of secondary metabolites, which are influenced by harvesting season and location [23]. Nevertheless, the observed antioxidant capacity of the *F. vesiculosus* extract is attributed to the phenolic compounds present, with phlorotannins standing out as the most abundant compounds in this alga. These compounds possess a high number of hydroxyl groups available to donate electrons or hydrogen atoms, thereby neutralizing free radicals that drive the oxidation of other molecules or biological structures [35].

## 3. Materials and Methods

### 3.1. Reagents and Plant Material

Folin–Ciocalteu (F–C) reagent, 2,2-diphenyl-1-picrylhydrazyl (DPPH), 2,4,6-tri (2-pyridyl)-1,3,5-triazine (TPTZ), gallic acid, and trolox were purchased from Sigma-Aldrich (St. Louis, MO, USA). Ethanol, hexane, water and methanol HPLC grade, glacial acetic acid, iron chloride hexahydrate, and sodium carbonate were purchased from VWR (Darmstadt, Germany). LC-MS grade methanol and water, acetonitrile, and sodium acetate were purchased from Merck KgaA (Darmstadt, Germany).

*F. vesiculosus* food-grade dry material was purchased from Bidah Chaumel (Murcia, Spain). It was ground to an average particle size of 0.8 mm and stored at −20 °C until extraction.

### 3.2. Extraction of Phenolic Compounds from F. vesiculosus Algae Using Sonotrode

Firstly, before extraction, *F. vesiculosus* dry and ground material were defatted by extraction with n-hexane in a ratio of 1:2 *m*/*v* under sonication for 20 s at 20% amplitude. After centrifugation, the pellet of defatted algae was allowed to dry at ambient temperature until stable weight. Then, 10 g of dry deffated *F. vesiculosus* material was extracted with 100 mL of an ethanol/water solution using a SONICS Vibra Cell ultrasonic processor (Sonics & Materials, Inc., Newtown, CT, USA) with a probe 13 mm in diameter. The extracts obtained for each run of the model were centrifuged at 14000 rpm for 15 min and the supernatant was transferred to 100 mL volumetric flasks and brought to volume with the appropriate solvent to proceed with the evaluation of the total antioxidant content using the Folin–Ciocalteu method.

### 3.3. Experimental Design

The optimization of the ultrasound-assisted extraction (UAE) of antioxidant compounds from *F. vesiculosus* was performed through a Box–Behnken design (BBD). It included 15 experimental runs in which the independent variables were ethanol % (*v*/*v*) (*X*_1_), extraction time (min) (*X*_2_), and amplitude (%) (*X*_3_), assayed at three levels (−1, 0, 1). Ultrasound was applied in a continuous mode through the extraction process.

Afterwards, total antioxidant content (TPC) was measured in the extracts (*n*= 3) in order to be considered as a dependent variable in response surface methodology (RSM). In this design, the dependent variable was fitted to a second order polynomial model equation (Equation (1)), in which *Y* means the response variable, TPC values; *Xi* and *Xj* are the independent factors that influence the response; and *β*_0_, *β_i_*, *β_ii_*, and *β_ij_* are the regression coefficients of the model (interception, linear, quadratic, and interaction terms). The adjustment of the model was evaluated through an analysis of variance (ANOVA) test, taking into account the coefficients, *p*-values, and lack of fit of the regressions.

Equation (1) is the second order polynomial equation for RSM.

(1) $$Y=\beta_{0}+\overset{3}{\underset{i=1}{\sum }}\beta_{i\;}X_{i\;}+\overset{3}{\underset{i=1}{\sum }}\beta_{ii}{X_{ii}}^{2}+\overset{2}{\underset{i=1}{\sum }}\overset{3}{\underset{j=i+1}{\sum }}\beta_{ii}X_{i}X_{j\;}$$

### 3.4. Total Phenolic Content Assay (TPC)

The F-C method was assayed to determine the TPC of the extracts [48]. Briefly, 400 μL of sample, standard, or 80% methanol blank was mixed with 800 μL of 10% (*v*/*v*) F-C reagent in test tubes. Then, 3200 μL of 700 mM Na_2_CO_3_ was added and incubated in the dark at room temperature for 2 h. Finally, the absorbance was measured using a Jenway spectrophotometer (Jenway, Felsted, UK) at 765 nm. Gallic acid was used as a standard and the results were expressed as mg gallic acid equivalents (mg GAE)/g dry seaweed.

### 3.5. HPLC-ESI-TOF-MS Analysis

The analyses were carried out on an ACQUITY UPLC system (Waters Corporation, Milford, MA, USA) coupled to an electrospray ionization (ESI) source operating in negative mode and a time-of-flight (TOF) mass detector (Waters Corporation, Milford, MA, USA). The separation of the compound was achieved through an ACQUITY UPLC BEH Shield RP18 column (1.7 mm, 2.1 mm × 100 mm; Waters Corporation, Milford, MA, USA) at 40 °C, using the gradient and mobile phases described by Martín García et al. [49]. The data were processed in MassLynx 4.1 software (Waters Corporation, Milford, MA, USA).

### 3.6. Antioxidant Capacity Assays

#### 3.6.1. Ferric-Reducing Antioxidant Power

The FRAP assay was executed based on Benzie and Strain’s protocol, with slight modifications [50]. The FRAP reagent was composed of a mixture containing 10 volumes of a 300 mmol/L acetate buffer (pH = 3.6), one volume of 10 mM TPTZ in acidic solution, and one volume of 20 mM FeCl_3_. Trolox was used as the standard. In each test, 3 mL of FRAP reagent was combined with 480 μL of either solvent (blank), standard Trolox, or sample solution in triplicate. Absorbance measurements were recorded at λ = 593 nm. Results were presented in terms of mmol trolox equivalents/g of dry extract (mmol TE/g d.w.).

#### 3.6.2. DPPH Assay

A modified approach to the method described by Brand-Williams [51] was applied to evaluate the extract’s radical scavenging capability. In brief, 100 μL of solvent (blank) or sample was combined with 3900 μL of 60 μM DPPH solution. The mixtures were incubated in the dark at ambient temperature for 30 min, after which absorbance was measured at 515 nm. The inhibition percentage (I%) was calculated using the following formula:

I% = [(A_DPPH_ − A_blank_) − (A_s-DPPH_ − A_s-blank_)]/(A_DPPH_ − A_blank_) × 100 (2)

where A_DPPH_ is the absorbance of the DPPH solution, A_blank_ represents the absorbance of methanol replacing DPPH, A_s-DPPH_ denotes the absorbance of the DPPH solution with sample, and A_s-blank_ is the absorbance of methanol with sample.

The EC_50_ value, defined as the extract concentration needed to achieve 50% DPPH radical inhibition, was determined from a calibration curve created with extract concentrations ranging from 0.5 to 61 μg/mL.

#### 3.6.3. Trolox Equivalent Antioxidant Capacity (TEAC) Assay

The TEAC assay followed the methodology of Re et al. [52]. A reaction between a 7 mM ABTS solution and 2.45 mM potassium persulfate over 16 h yielded the ABTS+ radical cation, detectable at 734 nm (λ = 734 nm). The absorbance was adjusted to 1.1 (± 0.02). Then, 2850 μL of the ABTS+ solution was mixed with 150 μL of either solvent (blank), standard, or sample. Results were expressed as μmol Trolox equivalents per gram of dry extract (μmol TE/g d.w.).

### 3.7. Statistical Analysis

The software Statistica v.7.0 package (StatSoft, Tulsa, OK, USA) was used for the experimental design, data analysis, and model building. The statistical significance of the model, lack of fit, and regression terms were evaluated based on the ANOVA. The results are expressed as the mean ± standard deviation (SD).

## 4. Conclusions

This study provides novel insights into the optimization of low-amplitude ultrasound-assisted extraction (UAE) using ethanol–water as a green solvent for phenolic compounds from *F. vesiculosus*, in comparison with existing studies focused on the extraction of phlorotannins, or that used other kinds of green solvents such as NADES. The optimal extraction conditions were determined to be a 40% ethanol/water mixture (*v*/*v*), an extraction time of 38 min, and an amplitude of 36%. These parameters achieved maximum efficiency in phenolic compound recovery and antioxidant activity, striking a balance between process effectiveness and environmental sustainability.

HPLC-ESI-QTOF-MS analysis tentatively identified 25 phenolic compounds, including 2 sulfated phenolic acids, 15 phlorotannins, 1 flavonoid, and 7 halophenols. Notably, 11 of these compounds, such as vanillic acid 4-sulfate, hydroxy tyroxol sulfate, sulfated diphlorethohydroxycarmalol, and lanosol sulfate, were reported for the first time in *F. vesiculosus*. This profiling highlights the chemical complexity and richness of secondary metabolites in this species, emphasizing its potential as a source of unique bioactive compounds. In addition, antioxidant assays (FRAP, DPPH, and TEAC) confirmed the high antioxidant capacity of the optimized extract, comparable to or surpassing values reported in previous studies.

In conclusion, this study provides an efficient and reproducible method for the extraction and characterization of bioactive compounds from *F. vesiculosus*, underscoring the potential of this brown alga as a natural source of antioxidants. The combination of advanced extraction and analytical techniques enhances its applicability in biotechnological, cosmetic, and food industries, promoting its valorization within a circular and sustainable economy framework.

These findings pave the way for future research aimed at validating the biological and functional properties of the identified compounds and optimizing their extraction for industrial-scale applications.

## Figures and Tables

**Table 1:** Natural and coded (in parenthesis) values of the extraction conditions and the experimental results for TPC obtained for the Box–Behnken experimental design.

Independent Factors				Dependent Factor
No	X1	X2	X3	TPC (mg GAE/g Dry Seaweed)
1	10 (−1)	2 (−1)	30 (0)	1.70 ± 0.12
2	10 (−1)	21 (0)	20 (−1)	1.68 ± 0.07
3	10 (−1)	21 (0)	40 (1)	2.52 ± 0.13
4	10 (−1)	40 (1)	30 (0)	2.39 ± 0.15
5	50 (0)	2 (−1)	20 (−1)	2.20 ± 0.09
6	50 (0)	2 (−1)	40 (1)	2.46 ± 0.20
7	50 (0)	21 (0)	30 (0)	2.63 ± 0.14
8	50 (0)	21 (0)	30 (0)	2.72 ± 0.16
9	50 (0)	21 (0)	30 (0)	2.70 ± 0.13
10	50 (0)	40 (1)	20 (−1)	2.62 ± 0.17
11	50 (0)	40 (1)	40 (1)	2.93 ± 0.09
12	90 (1)	2 (−1)	30 (0)	0.71 ± 0.06
13	90 (1)	21 (0)	20 (−1)	0.95 ± 0.11
14	90 (1)	21 (0)	40 (1)	1.43 ± 0.17
15	90 (1)	40 (1)	30 (0)	2.34 ± 0.18

X1-3: Ethanol/water (*v*/*v*), time (min.) and amplitude (%). TPC: Total phenolic content. GAE: Gallic acid equivalents.

**Table 2:** Estimated regression coefficients of the adjusted second-order polynomial equation (Equation 1) and analysis of variance (ANOVA) of the model.

Regression Coefficients	TPC (mg GAE/g d.w.)	
	Effect	*p*-Value
β0	1.9933	0.0000 *
Linear		
β1	−0.6492	0.0033 *
β2	0.9211	0.0016 *
β3	0.5372	0.0048 *
Crossed		
β12	0.4711	0.0112 *
β13	−0.1781	0.0710
β23	0.0276	0.6377
Quadratic		
β11	0.9069	0.0008 *
β22	−0.0005	0.9863
β33	0.1402	0.0328 *
R^2^	0.9942	
*p* model	0.0025 *	
*p* lack of fit	0.1945	

* Significant at α ≤ 0.05; 1 = ethanol-water ratio (*v*/*v*), 2 = time, 3 = amplitude.

**Table 3:** Optimal extraction and predicted and empirical values of the model are expressed as the mean ± SD (*n* = 3).

Parameter	Optimal Conditions
Ethanol (%)	40
Time (min)	38
Amplitude (%)	36
	Results
TPC predicted value (mg GAE/g d.w.)	3.01 ± 0.14
TPC empirical value (mg GAE/g d.w.)	2.89 ± 0.24
Coefficient of variation (%)	6.19
Yield of extraction (mg dry extract/g dry algae)	245.2 ± 9.1

TPC: Total phenolic content. GAE: Gallic acid equivalents.

**Table 4:** Characterization of phenolic compounds in *F. vesiculosus* extract using LC-ESI-QTOF-MS.

Peak No.	Retention Time (min)	*m*/*z* Exp. [M-H]^−^	*m*/*z* Calc. [M-H]^−^	Molecular Formula	Error (ppm)	Score	Fragments	Proposed Compound
Phenolic acids and derivatives								
1	1.101	246.9944	246.9912	C_8_H_8_O_7_S	13	92.23	203.0029 (-CO_2_), 123.0428 (-CO_2_, -SO_3_), 121.0294, 108.0213	Vanillic acid 4-sulfate
3	2.206	233.0119	233.0120	C_8_H_10_O_6_S	−0.4	85.44	153.0521 (-SO_3_), 123.0462	Hydroxy tyrosol sulfate
Phlorotannins								
2	1.73	497.072	497.072	C_24_H_18_O_12_	−1.1	99.99	479.0722 (-H_2_O), 353.0290(-1PGU, -H_2_O, -1), 339.0494, 230.0214 (-2PGU, -H_2_O, +1), 139.0032, 124.0742	Fucodiphloroethol
4	3.212	745.1027	745.1041	C_36_H_26_O_18_	−1.9	86.13	727.0961 (-H_2_O), 709.0927 (-2H_2_O), 585.0652, 477.0439 (-2 PGU, -16/-2 PGU, -H_2_O, +2), 453.0212, 353.0500, 267.0306, 229.0145, 165.0181	Fucophlorethol hexamer I
5	3.402	745.1021	745.1041	C_36_H_26_O_18_	−2.7	97.96	727.0924 (-H_2_O), 709.0813 (-2H_2_O), 621.0874 (-1PGU, +2), 603.0735 (-1PGU, -H_2_O. +2), 537.0646, 453.0399, 411.0307, 353.0197, 245.0097, 165.0190, 139.0041	Fucophlorethol hexamer II
6	3.5	621.0878	621.088	C_30_H_22_O_15_	−0.3	99.01	603.0768 (-H_2_O), 585.0658 (-2H_2_O), 559.0663, 477 (-1PGU, -H_2_O), 353.0272, 335.0200, 229.0137 (-3PGU, -H_2_O), 139.0058	Fucotriphlorethol isomer I
7	3.84	621.0879	621.088	C_30_H_22_O_15_	−0.2	99.96	603.0775 (-H_2_O), 585.0636 (-2H_2_O), 477.0457 (-1PGU, -H_2_O), 339.0500, 245.0087, 229.0143 (-3PGU, -H_2_0, +4), 139.0042	Fucotriphlorethol isomer II
8	5.401	745.1033	745.1041	C_36_H_26_O_18_	−1.1	82.9	727.0924 (-H_2_O), 601.0630 (-1PGU, -H_2_O), 583.0519 (-1PGU, -2H_2_O), 477.0485 (-2PGU, -16/-2PGU, -H_2_O, +2), 461.0493, 353.0289, 335.0193, 245.0081, 229.0141, 139.0038	Fucophlorethol hexamer III
9	5.645	745.1020	745.1041	C_36_H_26_O_18_	−2.8	95.71	727.0910 (-H_2_O), 709.0802 (-2H_2_O), 601.0621 (-1PGU, -H_2_O), 583.0531, 477.0448 (-2PGU, -16/-2PGU, -H_2_O, +2), 339.0496, 229.0128	Fucophlorethol hexamer IV
10	6.948	869.1188	869.1201	C_42_H_30_O_21_	−1.5	99.99	851.1092 (-H_2_O), 833.1029 (-2H_2_O), 725.0798 (-1PGU, -H_2_O), 707.0670 (-1PGU, -2H_2_O), 619.0766 (-2PGU, +2), 601.0629 (-2PGU, -16/-2PGU, -H_2_O, +2), 461.0506, 353.0299, 335.0214, 245.0108, 229.0146, 139.0029	Fucophlorethol heptamer I
11	7.233	869.1198	869.1201	C_42_H_30_O_21_	−0.3	81.55	851.1093 (-H_2_O), 833.0983 (-2H_2_O),725.0778 (-1PGU, -H_2_O), 707.0662 (-1PGU, -2H_2_O), 477.0472, 427.9290, 367.0085, 339.0498, 245.0094, 229.0132	Fucophlorethol heptamer I
12	8.057	993.1396	993.1362	C_48_H_34_O_24_	2.2	93.17	975.1302 (-H_2_O), 869.1177 (-PGU), 851.1154 (-PGU, -H_2_O), 603.0748, 461.0552, 229.0147	Fucophloretol octamer I
13	8.409	993.1406	993.1362	C_48_H_34_O_24_	4.4	81.64	975.1277 (-H_2_O), 849.0968 (-1PGU, -H_2_O), 635.0671, 601.0621, 461.0542, 353.0300, 247.0234, 229.0155	Fucophlorethol octamer II
14	8.607	933.1369	993.1362	C_48_H_34_O_24_	0.7	99.87	975.1270 (-H_2_O), 849.0989 (-1PGU, -H_2_O), 831.0840, 745.1002, 711.1008, 603.0814, 477.0471, 353.0313, 245.0103, 229.0139	Fucophlorethol octamer III
15	9.385	591.0070	591.0081	C_24_H_16_O_16_S	−1.9	100	511.0480 (-SO_3_), 385.0163 (-1PGU, -SO_3_, +1), 245.0124, 229.0139, 139.0042	Diphlorethohydroxycarmalol sulphate
16	9.509	1241.1735	1241.1683	C_60_H_42_O_30_	1.8	87.32	1223.1594 (-H_2_O), 1117.1472 (-1PGU, +2), 1099.1378 (-1PGU, -H_2_O, +2), 487.1113, 392.1327, 353.0309, 257.0666, 229.0140	Fucophlorethol decamer
17	9.745	1117.1542	1117.1522	C_54_H_38_O_27_	1.8	100	1099.1375 (-H_2_O), 975.1223 (-PGU, -H_2_O), 835.1108, 601.0684, 353.0303, 245.0073, 229.0148	Fucophlorethol nonamer
Flavonoids								
19	10.883	317.0298	317.0297	C_15_H_10_O_8_	0.3	100	178.9658	Myricetin
Halophenols								
18	10.618	376.8174	376.8330	C_7_H_6_O_6_SBr_2_	-	-	296.8598 (-SO_3_), 245.0122, 96.9615	Lanosol sulfate
20	10.883	476.7614	-	-	-	-	396.8675 (-SO_3_)	Dibromophenol sulfate
21	10.883	454.8676	-	-	-	-	374.8629 (-SO_3_)	Dibromophenol sulfate
22	10.932	154.9906	154.9900	C_7_H_5_O_2_Cl	3.9	100	-	2-Chlorobenzoic acid
23	10.957	332.9717	332.9721	C_16_H_8_O_4_Cl_2_	−1.2	99.57	154.9906, 111.0001	Dichlorophenol
24	10.957	392.9803	-	-	-	-	-	Trichlorophenol
25	10.957	488.9576	-	-	-	-	-	Tetrachlorophenol

**Table 5:** Antioxidant activity of *F. vesiculosus* extract.

Antioxidant Assay	Results
FRAP (mmol TE/g d.w.)	143.7 ± 5.8
DPPH (EC_50_ μg d.w./mL)	105.6 ± 3.2
TEAC (mmol Trolox/g d.w.)	189.1 ± 6.5

## Data Availability

Data are contained within the article.
